# Molecular mechanisms of action of stimulant novel psychoactive substances that target the high-affinity transporter for dopamine

**DOI:** 10.1042/NS20210006

**Published:** 2021-11-17

**Authors:** Michelle A. Sahai, Jolanta Opacka-Juffry

**Affiliations:** School of Life and Health Sciences, University of Roehampton, London SW15 4JD, U.K.

**Keywords:** addiction, dopamine transporter, molecular modelling, new psychoactive substances, psychostimulants, review

## Abstract

Drug misuse is a significant social and public health problem worldwide. Misused substances exert their neurobehavioural effects through changing neural signalling within the brain, many of them leading to substance dependence and addiction in the longer term. Among drugs with addictive liability, there are illicit classical stimulants such as cocaine and amphetamine, and their more recently available counterparts known as novel psychoactive substances (NPS). Stimulants normally increase dopamine availability in the brain, including the pathway implicated in reward-related behaviour. This pattern is observed in both animal and human brain. The main biological target of stimulants, both classical and NPS, is the dopamine transporter (DAT) implicated in the dopamine-enhancing effects of these drugs. This article aims at reviewing research on the molecular mechanisms underpinning the interactions between stimulant NPS, such as benzofurans, cathinones or piperidine derivatives and DAT, to achieve a greater understanding of the core phenomena that decide about the addictive potential of stimulant NPS. As the methodology is essential in the process of experimental research in this area, we review the applications of *in vitro, in vivo* and *in silico* approaches. The latter, including molecular dynamics, attracts the focus of the present review as the method of choice in molecular and atomistic investigations of the mechanisms of addiction of stimulant NPS. Research of this kind is of interest to not only scientists but also health professionals as updated knowledge of NPS, their modes of action and health risks, is needed to tackle the challenges posed by NPS misuse.

## Introduction

Drug misuse is a growing social and public health problem worldwide; it is harmful to the individual and society. According to the United Nations Office on Drugs and Crime (UNODC), approximately 275 million people used illicit substances in 2020; some 13% of those users have drug misuse problems [1] of whom a proportion will be dependent on the substances they misuse.

A range of misused substances can cause dependence at a high cost to both the individual and the community; opioid dependence is one side of the problem, while dependence on psychostimulants such as amphetamine and cocaine, continues to be a long-term issue worldwide. In 2019, approximately 27 million people used amphetamines and 20 million people used cocaine [1]. Amphetamine-like and cocaine-like drugs also constitute a proportion of new (or novel) psychoactive substances (NPS) having a potential of addiction and those will be in the focus of the present review. Addiction is a complex condition characterised by a destructive pattern of substance misuse that affects a person’s mental and physical health and distorts one’s social conduct and functioning at the level of community and society. While users of the classical stimulants, cocaine and amphetamine, are most often aware that these drugs are addictive, this is not the case for users of the less well-known NPS. People who reach for NPS as recreational drugs may not realise that repetitive use of novel psychoactive drugs may result in substance dependence leading to addiction. Regarding the terminology, and acknowledging there are multiple definitions, we use ‘dependence’ as an adaptive state with physical or physiological reliance on drugs that becomes unmasked during substance withdrawal, while ‘addiction’ means compulsive, out-of-control drug use, despite negative consequences [2].

The original definition of NPS proposed by the Advisory Council on the Misuse of Drugs (ACMD) in U.K. describes NPS as ‘psychoactive drugs which are not prohibited by the United Nations Single Convention on Narcotic Drugs or by the Misuse of Drugs Act 1971, and which people in the U.K. are seeking for intoxicant use’. NPS have been prevalent worldwide since 2005, yet their pharmacological characteristics remain largely unknown. In the U.K. they have been blanket-banned since 2016; before that, they were known as ‘legal highs’ and easily available online and in brick-and-mortar shops. NPS can cause acute and long-term health effects, including mental ill-health, dependence or even death. Some 950 new psychoactive substances have been identified at the global level since 2005. Thanks to the intensive research on NPS combined with public engagement and drug awareness campaigns, the number of NPS found globally seems to be recently stabilised at approximately 540 substances reported in 2019 while the number of NPS identified for the first time globally has gone down from 213 to 71 during 2013–2019. Although there are decline in NPS prevalence in North America and Europe, NPS use seems to be growing in low- and middle-income countries. Despite some positive reports on the decline in NPS use, NPS are associated with 10% of drug-related emergency hospitalisations in the EU and a mortality rate of 6% in U.K. As the national and international statistics tend to pool all types of NPS, it is impossible to tease out the extent of ill-health effects, including substance dependence, caused by stimulant NPS [1].

Of the synthetic NPS that have been reported until December 2020, the majority are stimulants, followed by synthetic cannabinoid receptor agonists, classic hallucinogens, synthetic opioids, sedatives or hypnotics and dissociatives [1]. In this review, we will focus on stimulant NPS, hence it is appropriate to recall the core characteristics of stimulant drugs. As demonstrated in experimental studies in rodents, stimulants normally increase dopamine (DA) availability in the brain, including the dorsal and ventral striatum, the latter comprising the nucleus accumbens (NAc) and being part of the mesolimbic pathway implicated in the reward-related behaviour [3]. This pattern is also observed in the human brain [4]. DA has been known to play an important role in reward learning and in the regulation of reward-related behaviours that leads to the moderation of hedonic stimuli [5]. It has been demonstrated that DA release in the NAc of the ‘reward pathway’ links with the perception of pleasure and reward; it also contributes to the biological underpinnings of drug dependence [6]. The dopamine theory of addiction has been widely recognised by neuroscience and pharmacology, but addiction is a complex phenomenon that implicates not only DA but also other neurotransmitters [7]. Nevertheless, for stimulant drugs, the role of dopamine in their addictive effects remains unchallenged.

The dopamine transporter (DAT) is directly implicated in the DA-enhancing effects of stimulants [7]. It has been widely accepted that both cocaine and amphetamine act at DAT, albeit via different mechanisms [8]. In brief, cocaine inhibits DA reuptake [8,9] while amphetamine enters the presynaptic neuron via DAT and displaces endogenous DA into the synaptic cleft [10]. In each case, the net effect is an increase in synaptic concentrations of DA and potentiation of dopaminergic neurotransmission in the brain, the mesolimbic/mesocortical pathways being of relevance to the behavioural change towards dependence and addiction. The molecular underpinnings of these mechanisms will be addressed in the course of this review.

While NPS interactions with DAT are thought to decide about addiction liability of these substances [11–14], other monoamine transporters (MAT), such as the norepinephrine transporter (NET) and serotonin transporter (SERT) also contribute to stimulant properties and thus to the addictive potential [15–17]. Many NPS have an affinity not only to DAT but also target NET and SERT [18,19].

DAT, NET and SERT belong to the neurotransmitter sodium symporter (NSS) family that consists of a group of regulatory proteins known as MAT; each transporter is heavily expressed within the mammalian brain [20]. These MAT play crucial roles in homoeostasis as well as neurotransmission associated with reward-seeking behaviour [20]. MAT have been associated with human brain disorders; abnormalities in the dopaminergic system have been implicated in not only drug addiction but also neurological disorders, such as Parkinson’s disease, attention deficit hyperactivity disorder (ADHD) and autism [21,22]. MAT have also become putative therapeutic targets for drug development against addiction.

Understanding the mechanisms of NPS interactions with their biological targets at the atomistic level contributes novel insights into the neurobiological effects of stimulant NPS; the present review will focus on the studies of the DAT in its interactions with stimulant NPS.

## Dopamine Transporter (DAT)

DAT plays a major role in the regulation of cognitive and emotional behaviours. As a major molecular target for misused psychostimulants, DAT is implicated in drug addiction, as mentioned above. DAT belongs to the SLC6A3 family of NSS that encode several Na^+^/Cl^−^-dependent neurotransmitter transporters, including the above-mentioned NET and SERT, and also transporters for γ-amino butyric acid (GABA) and glycine [23]. NSS proteins can couple the transport of Na^+^ downstream its concentration gradient with the upstream transport of the relevant substrate, DA in the case of DAT. Their role is to terminate the act of neurotransmission at chemical synapses, through reuptake of the neurotransmitter (e.g. DA) into the presynaptic cell that has released the neurotransmitter. DA released at the synapse then interacts with post-synaptic receptors; its excess is recycled by DAT expressed in the pre-synaptic membrane, DAT being abundant within the dopamine transmitting pathways in the mammalian brain, including the mesolimbic and mesocortical ones implicated in reward and addiction. DA taken up by DAT into the presynaptic terminal becomes either incorporated into presynaptic vesicles and reused in neurotransmission or oxidised through monoamino oxidases (MAO) to DA metabolites which no longer have neurotransmitter activity. Some of the extracellular DA escapes DAT and undergoes oxidation within the synaptic space.

**Figure 1:**
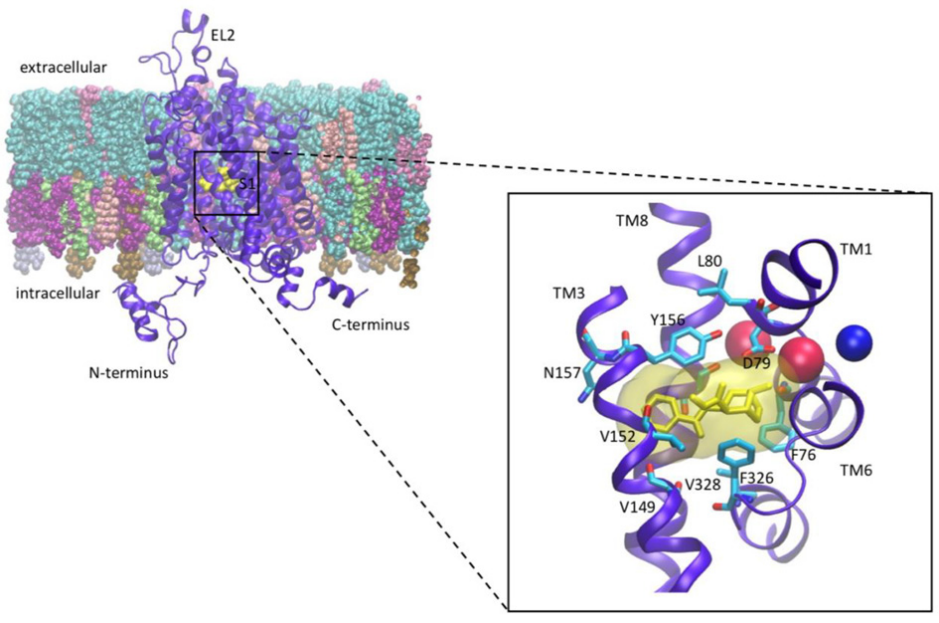
Structure of the DAT as illustrated in cartoon ribbons (in purple) imbedded in the physiological membrane The extracellular and intracellular sides as well as the N- and C-termini are clearly illustrated. The substrate binding pocket, S1 has been highlighted with a box and then zoomed out to show the positioning of cocaine in yellow (as an example NPS) in the binding site and important S1 binding residues. Sodium (red spheres) and chloride (blue sphere) ions around the S1 site are represented as well as the transmembrane domains (TM) 1, 3, 6, and 8, which are shown in blue; the other transmembrane domains and intra- and extracellular loops have been removed for clarity.

The substrate (DA) binding site is deeply buried in the molecule of DAT. It is known as the S1 site, which has been revealed by the X-ray crystallography of *Drosophila melanogaster* DAT (dDAT) and described by computational modelling [24–29]. While there is no crystal structure of the human dopamine transporter (hDAT) to date, computational modelling and simulations have been utilised to determine the conformational changes in the transmembrane (TM) domains of hDAT embedded in relevant physiological membranes that are involved in the allosteric coupling between ligand and ion transporter in DAT and the changes produced by specific psychostimulants [11–13,30–55,21,22,25]. The conformational changes have been quantified in terms of rearrangements of key residues, residue clusters and structural elements (e.g., helix ends, loops) that are important for regulating transporter mechanisms, and the interactions of DAT in the membrane environment during transport, signalling and trafficking. The details of the DAT structure with the extracellular and intracellular sides as well as the N- and C-termini are explained in Figure 1. The substrate-binding pocket, S1 and important binding site residues, as well as transmembrane helices and the location of the internal ions, are highlighted there.

In this article, we present an overview of research that concerns the molecular mechanism of actions of stimulant NPS at DAT. Research of this kind uses a range of methodologies including pharmacological, neurobiological and computational. We are providing a summary of the principles of these methods for the convenience of the reader who may not be familiar with them all, and we discuss their utility when applied to real cases for cocaine and amphetamine-type substances below.

## Methods used in NPS studies of relevance to the present review

### *In vitro* cell line binding assays

Transfected human embryonic kidney (HEK) cells 293 that express the hDAT or the human noradrenaline transporter (hNET) or the human 5-HT transporter (hSERT) are used to measure uptake inhibition at the MAT, including DAT [18,19,56,57]. Cells are grown under standardised conditions in commercially available cell culture media. For MAT binding assays, the medium is supplemented with pargyline as MAO inhibitor; DA, noradrenaline (NA), and 5-HT are normally used as substrates for DAT, NET and SERT respectively. Tritium-labelled radioligands, [^3^H]DA, [^3^H]NA and [^3^H]5-HT are used to trace the binding process while acting as radiolabelled substrates. Nonspecific uptake is controlled by high concentrations of relevant transporter blockers. Cells harvested from the cell culture, undergo rinses and preincubation before the assay that includes the radiolabelled substrate. Radioactivity retained in cells is quantified by a β counter. Saturation assays comprise increasing concentrations of the radiolabelled substrate. *K*_m_, IC_50_ and *K*_i_ values are calculated using relevant kinetic equations [58]. This technique has a high throughput and is widely used for screening new drugs, including NPS [18,19,56,57,59].

### *In vitro* fast cyclic voltammetry

Fast cyclic voltammetry (FCV) is an *in vitro* electrochemical method used to study drug effects on neurotransmitter release vs uptake in freshly harvested and physiologically active brain tissue. It records electroactive neurotransmitters, their basal and stimulated levels, with the use of reference, auxiliary, working (recording) and stimulating electrodes. The advantages of FCV are multiple, as precision electrodes implanted in specific brain regions of interest (ROI) facilitate the collection of data of high anatomical resolution and in real-time, with a millisecond temporal resolution [60–62]. The technique also offers sufficient chemical selectivity for neurotransmitters such as DA and other monoamines and avoids peripheral metabolic effects as well as blood–brain restrictions [9,63].

The details of FCV have been described previously [12,13,64]. In brief, a carbon fibre microelectrode is inserted under microscope guidance in the chosen ROI (e.g. NAc core) of a 400-µm-thick fresh brain slice maintained live under optimal *in vitro* conditions of temperature, pH and osmolarity. The bipolar stimulating electrode is inserted into the slice near the recording electrode. Auxiliary and reference electrodes are suitably placed in the slice chamber, away from the working electrode. The voltage that is applied triggers repeated oxidation and reduction of the neurotransmitter, which is monitored by the working electrode. Before the experiments, electrodes are calibrated in a standard DA solution; basal and stimulated DA levels are recorded during the experiment. The stimulus is standardised and pulses are programmed using specialist software. The current measured (e.g. every 100 ms) indicates the amount of electroactive neurotransmitter oxidised-reduced before and after a pharmacological manipulation, e.g. in response to an NPS that affects neurotransmitter uptake and release events [12,13,60,62,65].

### *In vitro* ligand binding at DAT – *in vitro* autoradiography

*In vitro* autoradiography (ARG) is a quantitative technique that tests the ligand–target interactions by means of radioactive ligands that bind with high selectivity and affinity to neurotransmitter binding sites (including transporters such as DAT interacting with substances such as NPS). Unlike tissue homogenate assays, *in vitro* ARG facilitates quantitative analysis of the regional distribution of neurotransmitter binding sites (targets) with a high anatomical resolution, as ARG uses tissue sections with undisturbed architecture [66,67].

ARG ligands are labelled with radioisotopes such as ^3^H or ^125^I, but also ^35^S or ^14^C. The radioligand used in ARG should have sufficient chemical stability, specific activity and the highest possible affinity and selectivity towards the binding site tested. High specific activity is necessary to ensure low concentrations of the ‘cold’, i.e. unlabelled ligand to reduce the non-specific binding. Having met these conditions, only trace amounts of radioligand are normally used, which reduces environmental and health risks. In the applications of relevance to the present review, we conduct ARG on serial frozen rat brain sections, 20-µm-thick, cut according to the rat brain atlas to represent the relevant ROIs; they can be stored at −80°C before the experiment.

### Competition binding (ARG)

Competition binding assays use increasing concentrations of a non-labelled (‘cold’) ligand that competes with the radioligand of choice which binds specifically at the active target site and remains at a constant concentration across the assay. The unlabelled substance, e.g. NPS tested, competes in concentration-dependent manner with the radioligand. The specific binding of the radioligand is quantified and the affinity of the tested substance (e.g. NPS) for the target is represented by the inhibitory constant IC_50_ which is consistent with the ability of the tested substance to inhibit 50% of the radioligand binding [68].

The details of the ARG procedure are described in our publications [11–13]. In brief, brain sections mounted on glass slides are incubated with the radioligand at optimal concentration, pH and temperature to achieve specific binding. The drug (NPS) tested is present at increasing concentrations to compete with the radioligand. Non-specific binding is measured in the presence of a cold (non-radioactive) ligand. The unbound ligand is removed by multiple rinses after the termination of the reaction. Dry slides undergo processing to visualise the radioactivity accumulated in the tissue. Digitalised densitometry can be used with calibration against commercially available quantitative radioactive standards. Image analysis using specialist commercially available systems provides quantitative data about the anatomical distribution of specific radioligand binding. ARG is a method of choice in studies on NPS [11–13,64,65].

### *In vivo* brain microdialysis to monitor DA release in response to drug

*In vivo* brain microdialysis is a sampling technique whereby a tiny tubular semipermeable membrane (similar to that used in the artificial kidney) is implanted in the brain ROI, and small molecules from the extracellular compartment cross the membrane by the laws of diffusion; the process is driven by the gradient of concentrations between the extracellular fluid and the medium used for perfusion, which is normally artificial cerebrospinal fluid (CSF) [69]. The osmotic power needs to be the same with the extracellular space to avoid water influx into the dialysis probe. The type of membrane that constitutes the microdialysis probe decides about the molecular weight cutoff for diffusible compounds such as DA. Artificial CSF is perfused through the probe at a constant rate of approx. 1 μl/minute (most common for rat brain, other than much earlier studies when higher flow rates were used). Sampling time depends on the research question asked and the sensitivity of the electrochemical chromatographic detection of dopamine and its metabolites; it varies between 5 and 20 min, more frequent sampling offers a better temporal resolution.

*In vivo* brain microdialysis is an invasive sampling technique as it involves surgical implantation of the probe, which for the brain means a stab wound. The surgical procedure uses stereotaxy under anaesthesia during which the microdialysis probe (or a guide for the probe) is permanently implanted and cemented to the skull. The animal is left to recover from anaesthesia for some 24 h during which time the blood–brain barrier reseals itself. A detailed description of the microdialysis procedure can be found in a number of original research publications [11,69]. Animals can be dialysed when free-moving such that behavioural observations can be coupled with neurochemistry, which is uniquely advantageous. Sample analysis for the contents of neurotransmitters such as DA and their metabolites is normally conducted using high-performance chromatography [70]. Brain microdialysis has been used in several studies on NPS [11,71–73].

### *In silico* molecular modelling of DAT with psychostimulants

The powerful computational approaches of molecular modelling and simulation have become widespread in drug research. Undeniably, such novel molecular–computational approaches to, for example, defining the therapeutic potential of NPS as substitution or maintenance treatment against classical psychostimulants, without the use of animals or their tissues, are advantageous and should become increasingly applicable in the future to a variety of therapeutic goals. Their framework fulfils the 3Rs (replacement, reduction and refinement) principles, through offering alternative methods to animal experimentation via computational approaches.

In particular, our research has been instrumental in combining experimental and computational methods to evaluate the structural changes produced by the binding of various NPS ligands to a rat (*Rattus norvegicus*) model of DAT (rDAT), and compare these with already ongoing work in hDAT models [11–13]. Mechanistic characterisation of these changes in rDAT is necessary as this is the primary animal model used in functional studies and pre-clinical testing of misused drugs.

We have also been able to link overall conformational changes of DAT bound to various ligands with observable pharmacological properties and physiological effects of drugs of abuse. An innovative combination of well-established computational modelling methods has been adapted to the complex transporter system to maintain the main features of approaches proven to be successful in other studies of similar systems [11–13,25,30–33,74].

The complex effects of investigated ligands, including the endogenous transported substrate, DA, and various NPS [64,65] on transport and signalling, require an understanding of both the mode of binding and the effect it has on the dynamics of the transporter and its interaction with the membrane environment. These include simulations performed on systems comprising the proteins embedded in atomistic models of biologically relevant membrane bilayers immersed in an aqueous solution of ions at physiological concentrations, see [11–13,25,30–33,74] for a description of simulations of similar systems.

**Figure 2:**
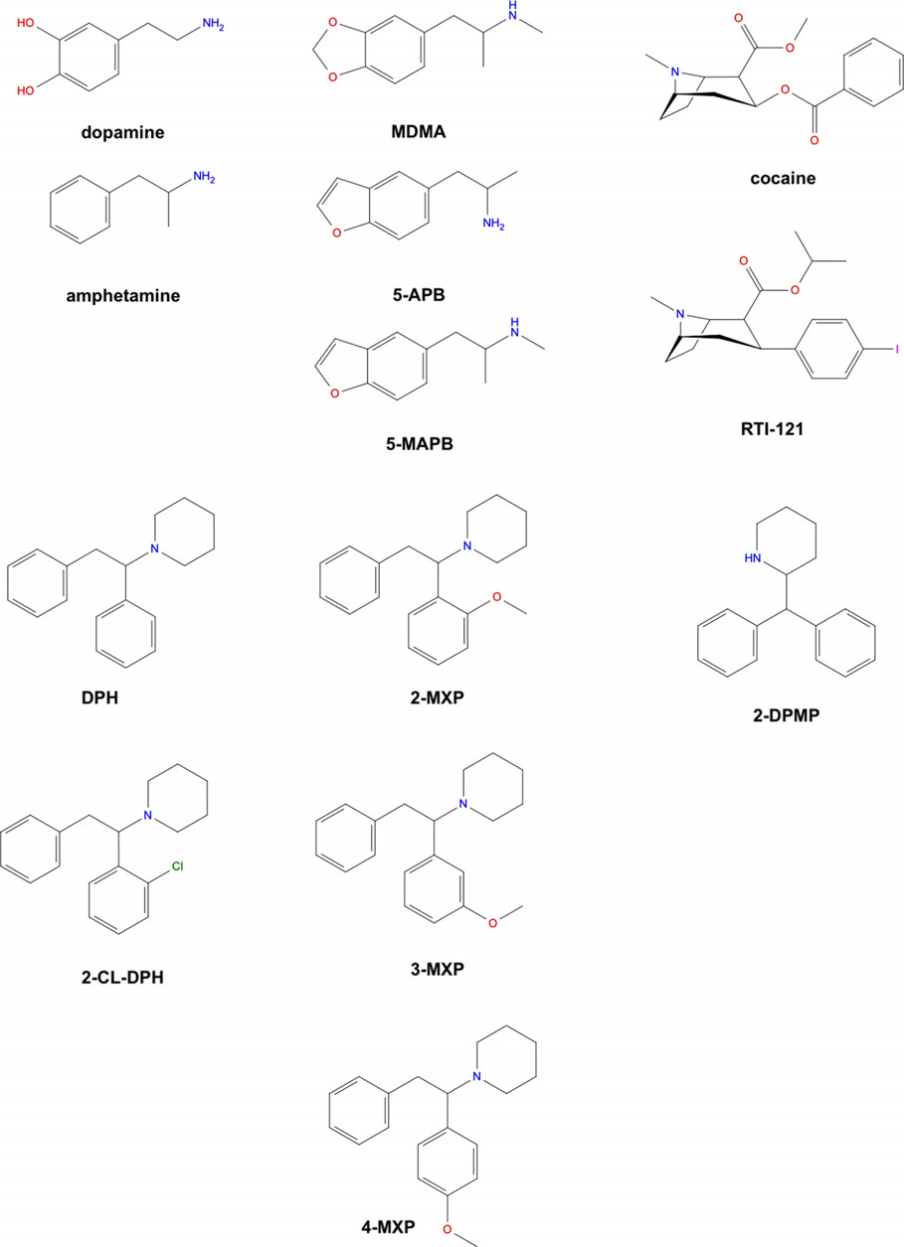
Molecular structures of various substrates and stimulants previously studied: dopamine, amphetamine, 3,4-methylenedioxymethamphetamine (MDMA), 5-APB (‘benzofury’), 5-MAPB, cocaine, RT1-121, diphenidine (DPH), methoxphenidine (2-MXP/MXP), 2-Cl-DPH, 3-MXP, 4-MXP and 2-diphenylmethylpiperidine (2-DPMP)

The main steps we have undertaken in our studies [11–13] are:

*Model development and validation*: We have constructed, tested and compared a novel structural model of the rDAT with the inclusion of the N- and C-terminal regions as well as refined loop regions similar to current and previous work [43,44]. The homology models of hDAT and rDAT are based on the X-ray crystal structures available for the dDAT [26–28]. Previously constructed hDAT models, which have been based on the dDAT crystal structure and on the bacterial homologue LeuT, a leucine transporter, have been used successfully to analyse the mechanisms of disease-related mutants in humans [21]. A comparison of these models based on the two templates showed that the critical regions of the binding site and ion binding sites are strikingly similar [21,22]. Other studies have utilised homology modelling in the absence of a crystal structure of hDAT with similar results [14,37,39,45,46,48,75].
*Computational docking*: We have also employed molecular docking to place the substrate DA and screened NPS compounds (Figure 2) into the rDAT model and compared it with similar docking studies in hDAT [11–13,31,76]. Docking as a molecular modelling technique to predict the position and orientation of ligands in the binding site of their target protein has also been utilised in other studies of NPS [14,32,75,77,78,33,37,39,40,44,46,48,52].
*Computational simulation:* We and others [11–13,37,41,45,46,48,79] have observed that conformational changes emerging over long-scale simulations, and free-energy calculations, can indicate the structural and dynamic elements of the mechanisms governing the ligand interactions and pharmacological effects of NPS on DAT. Inferences were probed using comparisons among various structural families of NPS which are expected to elicit structure-related structural and dynamic consequences of binding.

Previous studies that have also combined various experimental methods, such as mutagenesis and cross-linking experiments with *in silico* DAT structure–function studies have gone on to characterise the binding site for various NPS stimulants, such as the S1 binding site overlap of various stimulants like cocaine, cocaine analogues, benztropine (BZT) and its analogues and the substrate DA [32,33]. These studies were first to demonstrate the molecular basis for the competitive inhibition of dopamine transport by cocaine. Medicinal chemistry efforts combined with *in vitro* binding experiments and molecular dynamics have also revealed the structural requirements for different binding modes of stimulants [46,52,53]. Structure–activity relationships (SARs) combined with mutagenesis have revealed the side-chain contributions at the DAT binding site can be different for uptake inhibitors [53] such as BZT compared with cocaine, methylphenidate and mazindol. Further studies [34,35] have revealed the pathways for substrate release from the outward-facing towards the inward-facing conformation, often described as the ‘alternating access’ mechanism [80] and pharmacological characterisation and selectivity at MATS for amphetamine-like cathinones [43].

### Amphetamine-like NPS

NPS with amphetamine-, aminoindan- and benzofuran basic chemical structures have recently emerged for recreational drug use but detailed information about their psychotropic effects and health risks is often limited. At the same time, it appeared that the pharmacological profiles of these NPS resemble those of amphetamine or 3,4-methylenedioxymethamphetamine (MDMA) (Figure 2), which acts at DAT like amphetamine but also shows a significant affinity to SERT. Analysis of pharmacological profiles and *in vivo* effects and toxicity suggests that abuse liability for amphetamine-like NPS may be higher than for MDMA-resembling NPS, but that the risk for developing the life-threatening serotonin syndrome may be increased for MDMA-like NPS [81], especially in terms of their interactions with SERT and not only DAT or NET, as demonstrated by means of *in vitro* analysis in cell lines [18].

According to the UNODC, the amphetamine-type stimulants (ATS) are a group of substances composed of synthetic stimulants, controlled under the Convention on Psychotropic Substances of 1971, which includes amphetamine, methamphetamine, meth-cathinone and the ‘ecstasy’-group of substances (MDMA and its analogues) [1]. Amphetamine and ATS obstruct neurotransmitter reuptake and typically induce transporter-mediated efflux of neurotransmitters, causing an increase in neurotransmitter concentrations in the synaptic cleft. We focus here on studies that have combined computational and neurobiological methods to investigate ATS and have been key to our current understanding of the structure–function relationships between specific amphetamine-like NPS and DAT, that is preferentially inhibited by these compounds [82].

Among the ATS are benzofurans, which are phenethylamine derivatives and related to MDMA and 3,4-methylenedioxy-amphetamine (MDA), both currently classified as Class A drugs in U.K. We have previously examined 5-APB (commonly known as ‘benzofury’), a representative benzofuran (Figure 2) [64]. We measured 5-APB effects on the DAT using FCV and *in vitro* ARG in rat brain tissue, the latter not only for DAT but also for serotonin 5-HT2 receptor implicated in the hallucinogenic response [83,84]. Our findings suggested potential addictive and hallucinogenic properties of 5-APB, and amphetamine-like effects on DAT, as identified with FCV. Using FCV and *in vitro* ARG, we studied stimulant characteristics of cathinones, including mephedrone, a highly prevalent NPS in U.K. Based on FCV data, its actions at DAT were again similar to those of amphetamine [65].

To explore the core molecular and atomistic mechanisms of stimulant NPS, we studied the interactions between another benzofuran NPS and DAT using 5-MAPB as an example (Figure 2) [13]. Like in the case of 5-APB, *in vitro* ARG found that 5-MPAB can displace the tritium-labelled cocaine analogue RTI-121 and bind to the DAT, and elicits amphetamine-like effect on DAT, as identified with FCV. Additionally, using molecular modelling and *in silico* approaches, we explored a binding mode for 5-MAPB and, more broadly, the structural context for NPS effects at DAT as the molecular target of stimulants. Briefly, the homology model of rDAT was used in docking studies for 5-MAPB, amphetamine, 5-APB, MDMA, cocaine and RTI-121 (Figure 2). The findings confirm that these compounds share a binding site at the centre of rDAT and is located in a position corresponding to the primary substrate-binding site (S1) for the substrate DA, as seen in previous modelling studies [21,22,30–33]. Molecular dynamics simulations revealed a spontaneous transition of rDAT from the occluded to the inward-facing conformation and concomitant release of Na^+^ ion from the functional Na2 ion site. This has been shown previously to be induced by PIP_2_ lipid-mediated interactions between the N-terminal domain and the intracellular loop 4 (IL4) of DAT [30,31]. This study demonstrated the amphetamine-like mechanism of action at the molecular level. The findings, which warn of addictive potential typical of stimulants, are of relevance to the health risks linked with NPS use.

Using both computational and neurobiological methods – molecular modelling, FCV and ARG, we also investigated interactions with DAT across a range of ketamine-like dissociative NPS (Figure 2) [12]. With FCV we were able to estimate the stimulant profile of diphenidine (DPH) and its methoxylated derivative 2-methoxydiphenidine (methoxphenidine, 2-MXP/MXP), revealing that DPH is more similar to that of amphetamine, with increased DA efflux as a result of its binding to DAT, whereas MXP had no significant effect on either DAT binding or evoked DA efflux. The present study revealed the unknown addictive potential of a prevalent dissociative NPS, diphenidine. Despite their structural similarities, dissociative NPS exhibit different stimulant profiles, and thanks to the *in silico* approaches we explained why subtle structural differences result in such varied stimulant features of relevance to the risk of addiction in users of these NPS. We explored this class of compounds that share the core of the 1,2-diarylethylamine structure and an ethylamine nucleus with aromatic substitutions by molecular dynamics and alchemical free energy simulations. Structural rearrangements in DAT, when bound to DPH, show evidence of an outward-facing conformation, specifically the rearrangement of the extracellular gates, a well-known conformation adopted by inhibitors like cocaine. In contrast, when 2-MXP is bound to DAT, we find evidence of inward-facing conformation of DAT, such as the spontaneous release of Na2 and the rearrangement of the intracellular gates. Although this is a conformation adopted by many substrate releasers like amphetamine and 5-MAPB, 2-MXP does not appear to be a DAT inhibitor nor does it reveal reverse transport at the concentrations used in this study [12]. Free energy calculations also revealed from the predicted binding free energies that the 2-methoxy (–CH_3_O) group substitution on MXP is unfavourable, less potent at DAT when compared with DPH, which can be classed as the scaffold structure. Longer atomistic simulations are needed to resolve the rearrangements seen for 2-MXP as well as the other derivative structures 2-Cl-DPH, 3-MXP and 4-MXP (Figure 2) that also show stabilisation of the inward-facing conformation of DAT. Our study suggests that DPH can have addictive liability, unlike MXP, despite the chemical similarities of these two NPS [12].

What these studies [11–13] have shown and corroborated by previously published computational studies [31,37,47,85], is that the ATS-type substrates bind to the DAT S1 site, similarly to the substrate dopamine [31,85], and then continue to proceed deeper into the protein, committed to releasing into the intracellular vestibule and avoiding release at the extracellular side. The overall conformational changes observed by simulations show that rearrangements of the TM helices, alongside changes in the side-chain rotations and dislocation of the Na^+^ from the Na2 site, promotes an influx of water from the intracellular side and allows the protein to transition into this inward-facing conformation.

Docking and simulation studies supported by binding experiments show that the ATS-type stimulants bind at the S1 site, displacing the cognate substrate dopamine and binding in an almost identical geometry with the neurotransmitter dopamine [12,13,37], suggesting a similar mechanism of action to DA. While this is true for most ATS-type stimulants, we [12] and others [44,48] have also shown how small modifications to the scaffold structure of ATS-type stimulants can lead to varied flexibility in the binding site, where these analogues can inhibit uptake as well as induce release at DAT. This was supported by the quantitative structure–activity relationship (QSAR) [48,86,87], which is a method that has been used to examine the effects of altering the chemical structure of a given drug molecule on biological responses for ATS-type stimulants, but more recent cocaine-like ones which will be discussed in the next section.

### Cocaine-like NPS

DAT X-ray crystallography structures have been resolved in the outward-facing conformation [26–29] and have been the starting point for homology modelling and subsequent docking and simulation studies of cocaine and cocaine-like stimulants. It has been well documented that these compounds prevent the inward transition of TM helices, which opens the intracellular gates and subsequentially closes the extracellular gates. Instead, these inhibitors arrest DAT in the outward-facing conformation, primarily because of their molecular size, which hinders the transitions that we have seen for DA and amphetamine-like stimulants above [32,33,37].

Given the high addiction potential of cocaine and the upward prevalence of cocaine use [1], we have also recently investigated the mechanism of action of cocaine-like NPS, choosing as an exemplar 2-DPMP (Figure 2), known by its users as ‘Ivory wave’, in which we applied *in vitro* ARG, molecular dynamics as well as *in vivo* microdialysis of the NAc shell as the ROI of relevance to the mechanism of addiction [11]. 2-DPMP is a pure DAT inhibitor as it was previously shown to stimulate evoked DA efflux in NAc brain slices to a greater extent than cocaine [60]. *In vitro* binding by ARG revealed that 2-DPMP can potently displace the selective DAT-radioligand [^125^I]RTI-121 in the rat NAc and dorsal striatum. *In vivo* microdialysis also showed that 2-DPMP elicited a dose-dependent increase in extracellular DA in the brain’s ‘reward pathway’ in freely moving rats, consistent with the fast-acting potent stimulant profile. Molecular dynamics simulations also revealed contrasting conformational changes of DAT for the inhibitors compared with releasers. Specifically, structural rearrangements of the transporter toward an inward-facing conformation were observed for amphetamine, while cocaine and 2-DPMP binding inhibits DA transport forcing DAT to remain in the outward-facing conformation, similar to what was observed for DPH above [12].

In the early days of QSAR-based screening, models were used to design ligands due to the lack of crystal structures of the human NSS/SLC6 protein family. Nowadays, with the abundance of 3D structural information, ligand-based QSAR has become a much faster way to predict the correlation between modifications in stimulant chemical structures and binding affinities. We have already seen how small modifications can change the binding mechanism for amphetamine-like stimulants [12,48], and likewise, QSAR has been instrumental in differentiating stimulants as releasing agents or cocaine-like reuptake inhibitors by identifying key structural features, such as the nature and size of the substituent modification [88,89] and stereochemistry [54,77], all-important in predicting the biological activity for NPS like benzodiazepines and transporter selectivity [88,90].

### Impact of NPS research

Only a small proportion of NPS have been researched in preclinical studies of which some have influenced changes to the law in U.K. and EU [91]. One of our studies [64] is among those as it was used by the ACMD in their recommendation for Class B substance classification for Benzofurans [92], which led to the Amendments to the Misuse of Drugs Act 1971 in 2014. Research findings of this kind would also be useful for drug policy that does not criminalise substance misuse but considers it as a public health issue.

Preclinical research on NPS can also have an impact on practice as it is the case with our work [64] that was included in research-based evidence of the harmful effect of benzofurans by the Novel Psychoactive Treatment UK Network ‘NEPTUNE’ which provides clinical guidance to practitioners in U.K. and EU [93]. In addition, practitioners of charities that support people with addiction to drugs, use knowledge transfer from academic research to improve their staff training, practice and service delivery [94].

In terms of potential future impact, our research [11–13,64,65] reveals the molecular mechanisms governing the interactions between DAT and NPS and explains how their pharmacological properties can pave the way towards studies of other NSS proteins and can be used in the design of new therapeutic drugs to treat substance addiction apart from informing decisions by government agencies in charge of drug policy.

## Conclusions and future directions

Our research [11–13] has documented the meaningful progress achieved from combined computational and experimental studies. This is demonstrated not only by the nature and impact of the results obtained thus far but also in the questions and hypotheses generated for future work. We propose a new perspective for the computational studies that address not only the NSS proteins but also the interactions of their structural elements with other molecules and membrane environments that integrate them into cell physiological processes. To support these innovative perspectives, we have used combinations of newly developed computational methods and algorithms with established methods of computational modelling and simulations. Such studies have shed new light on the mechanisms of NSS proteins, in particular the DAT where we have observed structural and dynamic properties as well as dynamic mechanisms involved in both function and dysfunction of NPS bound to DAT.

By utilising computational modelling, we have shown that an increasingly integrative structural context can improve our understanding of the functional mechanisms of the neurotransmitter transporters. This is especially important when the mechanisms of interest underlie observable pharmacological properties and physiological effects of drugs of abuse and make these NSS proteins both a target for the design of therapeutic drugs, and a key element in (i) the determinants, (ii) the effects and (iii) the undesirable consequences, of substance abuse.

By aiming for a mechanistic characterisation of DAT, in different functional states of the proteins (e.g, as induced by drugs of abuse), based on a hypothesis that functional roles of segments like the N- and C-terminal depend on (i) specific conformations they adopt in different functional states and (ii) modulation of these conformations by cellular processes, we have been able to characterise different NPS based on their structural interactions with DAT. This is clear from our recent study where structural rearrangements of the transporter toward an inward-facing conformation were observed for releasers like amphetamine, while the inhibitors – cocaine and 2-DPMP – binding prevents DA transport forcing DAT to remain in the outward-facing conformation [11].

There has been an ongoing examination of the functional and pharmacological roles of MAT oligomerisation. Oligomerisation has been implicated in the efficient trafficking of newly formed proteins from the endoplasmic reticulum to their expression on the plasma membrane [95,96]. In addition to oligomerisation playing a part in transporter-mediated monoamine efflux (i.e., reverse transport or release), psychostimulants such as methamphetamine and amphetamine have been shown to influence transporter oligomerisation [97]. Although much of the underlying mechanisms remain vague, this is in contrast with other studies where amphetamine and dopamine were shown to disrupt oligomer formation in DAT [98,99], while cocaine had no effect on oligomerisation [98]. However, it is interesting that cocaine-induced formation of DAT oligomers can be reversed with amphetamine [100]. In general, there is still much to learn about MAT oligomerisation and the amphetamine-induced changes may reflect a conformational change that is dependent on the lipid environment, which can then be studied computationally [97].

We are also applying similar methodologies to study NPS stimulant effects, with dynamic analysis of various compounds and their analogues interacting with the other members of the MAT, NET and SERT. Investigations of a large repertoire of stimulant compounds bound to hSERT, hDAT and hNET could provide a pharmacological characterisation of selectivity when combined with functional studies. Having 3D structural information such as the determination of the crystal structure of hSERT [101] will aid in this endeavour. Additionally, machine learning-based QSAR modelling and molecular simulations, working in concert with traditional functional studies to define the stimulant properties of NPS and effectively their addictive potential, are advantageous and should become increasingly applicable in the future. This was recently shown to be successful in identifying a pair of DAT inhibitors having opposite binding affinity trends at DAT and human *ether-a-go-go* (hERG) [102], which can then be exploited rationally to design DAT inhibitors that could potentially be used in a treatment regimen for psychostimulant use disorders.

Ultimately, the goal is to assess the addictive liability of NPS and inform about the health risk related to their use. As the recreational use of NPS poses new challenges for healthcare, health professionals should be informed about new research findings concerning NPS, including their modes of action, and medical and psychological risks, as Schifano et al. (2021) postulate [103]. Moreover, DAT ligands might be useful as substitution or maintenance treatments for psychostimulant misuse and as anti-addiction therapy [104] or as treatments for ADHD or even some forms of depression [105].

## Data Availability

Data sharing is not applicable to the paper as it is a review. All data discussed from previous studies have been referenced appropriately.

## Abbreviations

ACMD: Advisory Council on the Misuse of Drugs
ADHD: attention deficit hyperactivity disorder
ARG: autoradiography
ATS: amphetamine-type stimulant
BZT: benztropine
DAT: dopamine transporter
dDAT: *Drosophila melanogaster* DAT
DPH: diphenidine
FCV: fast cyclic voltammetry
hDAT: human dopamine transporter
hNET: human noradrenaline transporter
hSERT: human 5-HT transporter
MAO: monoamino oxidase
MAT: monoamine transporter
MDMA: 3,4-methylenedioxymethamphetamine
MXP: methoxphenidine
NAc: nucleus accumbens
NET: norepinephrine transporter
NPS: novel psychoactive substance
NSS: neurotransmitter sodium symporter
QSAR: quantitative structure–activity relationship
rDAT: rat (*Rattus norvegicus*) model of DAT
ROI: region of interest
SERT: serotonin transporter
TM: transmembrane
UNODC: United Nations Office on Drugs and Crime
